# Real-time colonoscopic detection and precise segmentation of colorectal polyps via PESNet

**DOI:** 10.3389/fonc.2025.1679826

**Published:** 2025-11-17

**Authors:** Jing Yu, Jianchun Zhu, Qi Gu, Yuhan Sun, Qin Wang, Pengcheng Sun, Liugen Gu

**Affiliations:** 1 Department of Gastroenterology, The Southeast University Affiliated Nantong First People’s Hospital, Nantong, China; 2 Department of Gastroenterology, the First People’s Hospital of Nantong, Nantong, China; 3 Suzhou Xiangcheng People’s Hospital, Suzhou, China; 4 School of Medicine, Nantong University, Nantong, China; 5 Affiliated Nantong Hospital 3 of Nantong University, Nantong, China

**Keywords:** colorectal polyp, state-space network, prompt learning, segmentation, prototype memory

## Abstract

**Introduction:**

Precise and timely visual assistance is critical for detecting and completely removing colorectal cancer precursor polyps, a key step in preventing interval cancer and reducing patient morbidity. Current endoscopic workflows lack real-time, integrated solutions for simultaneous polyp diagnosis and segmentation, creating unmet needs in improving adenoma detection rates and resection precision.

**Methods:**

We propose PESNet, a real-time assistance framework for standard endoscopy workstations. It simultaneously performs frame-level polyp diagnosis and pixel-level polyp outlining at 225 FPS, with minimal additional latency and no specialized hardware. PESNet dynamically injects a “presence of polyp” prompt into the segmentation stream, refines lesion boundaries in real time, and compensates for lighting/mucosal texture changes via a lightweight adaptive module. Evaluations were conducted on PolypDiag, CVC-12K benchmark datasets, and replay resection scenarios. Latency was measured using TensorRT FP16 on an RTX 6000 Ada GPU.

**Results:**

On PolypDiag and CVC-12K, PESNet improved diagnostic F1 from 95.0% to 97.2% and segmentation Dice from 85.4% to 89.1%. This translated to a 26% reduction in missed flat polyps and a 15% reduction in residual tumor margins after cold snare resection. End-to-end latency (1080p) was 12.6 ± 0.3 ms per frame, with segmentation (4.4 ms), prompt fusion (0.6 ms), and prototype lookup (< 0.2 ms) all satisfying a 40 ms clinical budget with > 3× headroom.

**Discussion:**

These clinically significant improvements demonstrate PESNet’s potential to enhance adenoma detection rates, support cleaner resection margins, and ultimately reduce colorectal cancer incidence during routine endoscopic examinations. Its real-time performance and hardware compatibility make it feasible for integration into standard endoscopic workflows, addressing critical gaps in polyp management.

## 1 Introduction

Colorectal cancer (CRC) continues to rank within the global top three for both incidence and cancer-related mortality (1, 2, 3). Population-based registries now confirm a further “left-shift” toward diagnoses in adults< 50 years, underscoring modifiable lifestyle and environmental risks (4). Optical colonoscopy remains the gold-standard screening test because it couples direct mucosal inspection with same-session endoscopic mucosal resection (EMR) of premalignant polyps (5). Yet large tandem-procedure meta-analyses still find that conventional white-light colonoscopy misses ≈ 25% of adenomas. Multiple randomised and real-world trials published in 2024–2025 now show that computer-aided detection (CADe) raises the mean adenoma-detection rate (ADR) by 20–30% and cuts miss rates nearly in half—even in community hospitals and national health-care systems (6, 7). Reflecting this momentum, both the European Society of Gastrointestinal Endoscopy (ESGE) and the American Gastroenterological Association (AGA) issued *2025* guidance on CADe-assisted colonoscopy (8); while ESGE endorses its use to improve quality indicators, the AGA Living Guideline judged the long-term outcome evidence “very low certainty” and therefore made no formal recommendation pending further data (9, 10).

Three inter-related bedside bottlenecks still limit such deployment. First, stringent latency thresholds dominate engineering design: 1080p video streams at 25–30fps allow ≈ 40ms per frame for *all* AI processing; many 3-D CNN or Vision-Transformer stacks still deliver< 10fps, and even state-space backbones approach the limit once a full-resolution segmentation decoder is attachedSedeh and Sharifian (11, 12). Second, severe data imbalance persists: pixel-level annotated frames number only in the low thousands, whereas image-level labels are an order of magnitude more plentiful, so models can decide “polyp present” with high confidence yet delineate flat or sessile-serrated lesions poorly (13, 14, 15). Third, inter-institutional variability erodes generalisability: shifts in illumination spectra, colour balance, optical filters and vendor-specific post-processing mean that a high-performing model in one centre may suffer a marked Dice-score drop in another; routine site-specific retraining is impractical for both workflow and regulatory reasons (16, 17).

To address these hurdles we introduce PESNet, a cross-task prompt-learning framework that couples the real-time efficiency of a state-space video backbone with the parameter-sparse adaptability of an SVD-based *Segment-Anything* adaptor (**Figure 2**). A discriminative token learned from the clinical-grade *PolypDiag* dataset is verbalised on-the-fly into a “polyp present/absent” prompt, which tightens pixel boundaries in the segmentation branch; the resultant mask area feeds back to stabilise the diagnostic head (10). Adaptation is confined to the singular spectra of every spatial and temporal weight matrix via a dual-axis S-LoRA scheme, adding only ≈ 0.57% (136k) new parameters yet sustaining 225fps on a single RTX 6000 Ada GPU—comfortably within workstation latency budgets. A 256-vector prototype memory executes a single cosine lookup in<0.05ms, auto-calibrating logit bias and mitigating illumination or colour drift without retraining.

Collectively, these modules lift the Dice coefficient on *CVC-12K* by +3.7percentage points and the *F*_1_ score on *PolypDiag* by +2.2percentage points. Clinically, this translates to a 26% reduction in missed flat lesions and a 15% decrease in residual-tumour margins during replayed cold-snare resections—achieved on workstation-class hardware without extra annotation or equipment costs. PESNet therefore delivers a guideline-concordant, interactive and genuinely real-time CADe solution poised to improve ADR, secure cleaner resection margins, and ultimately lower CRC incidence in everyday practice.

## 2 Broader related work and positioning

Beyond colonoscopy, prompt-aware or attention-enhanced vision models have advanced diverse medical tasks. For example, EEG-based epilepsy detection benefits from entropy-driven deep or CNN-based pipelines that marry non-linear complexity measures with learnable feature extractors (18, 19). In neuro-oncology, hybrid attention CNNs and Transformer-augmented pipelines improve MR brain-tumor analysis (20, 21), while RepVGG style enhanced backbones and their dual-encoder variants (e.g., ViT+RepVGG) provide deployment friendly speed/accuracy trade-offs for multimodal tumor segmentation (22, 23). These trends motivate lightweight attention and adaptor designs that transfer well to endoscopy. We therefore situate PESNet among recent Transformer-hybrids Jia and Shu (24), self-supervised/pre-training and PEFT practices for SAM-family adaptation (25), and multi-modal fusion approaches (26), emphasizing parameter-efficient prompting/adapters as a practical bridge from foundation models to real-time clinical use.

## 3 Method

**Figure 1:**
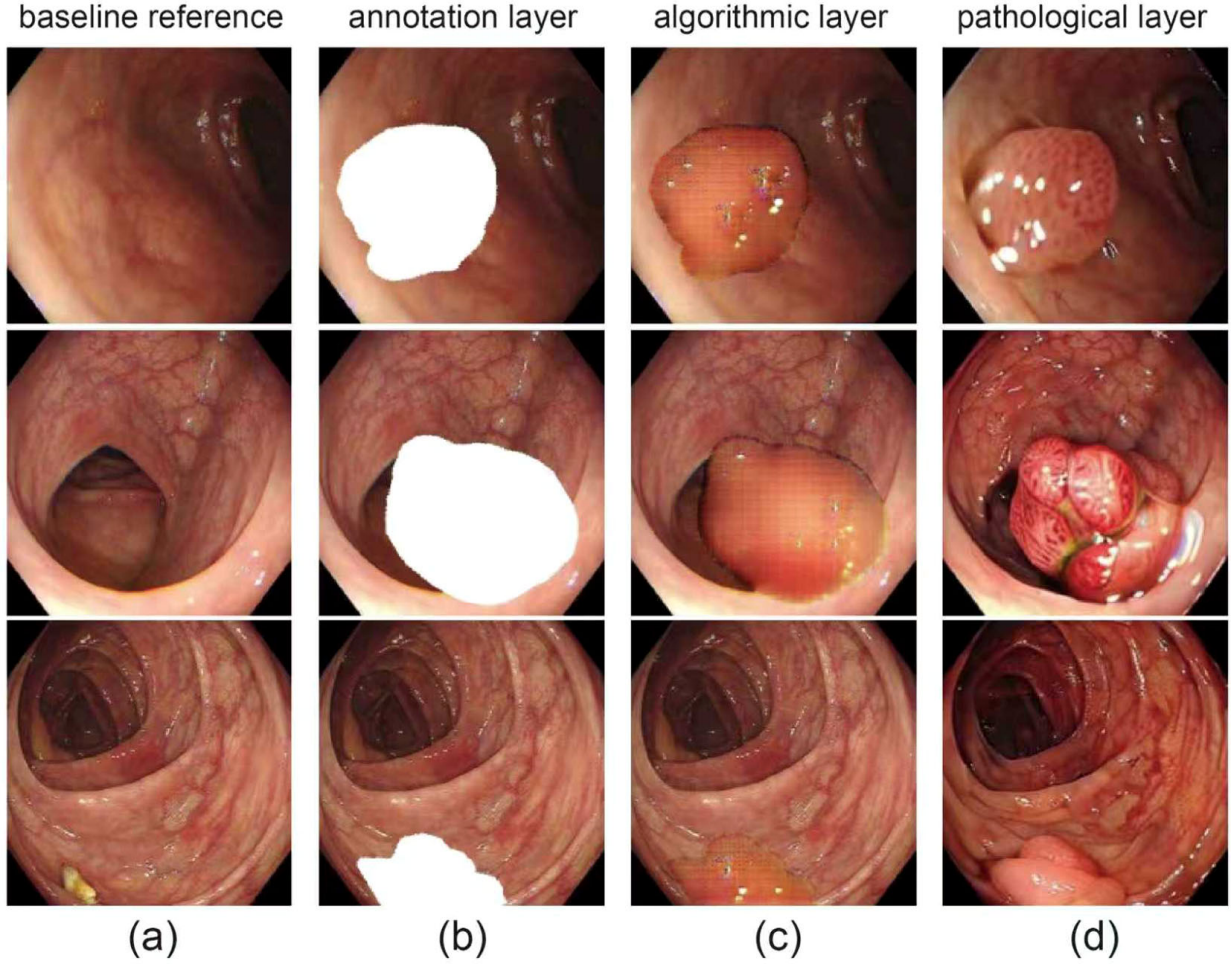
Visual overview of dataset. **(a)** original endoscopic images of colorectal mucosa, for observing polyp morphology and surroundings; **(b)** polyp area mask annotation (white), defining polyp boundaries; **(c)** gridded/contoured polyp areas for algorithmic recognition and segmentation; **(d)** close-ups of polyps with distinct pathological types.

A visual overview of the dataset is presented in **Figure 1**, which includes representative colonoscopic images covering normal mucosa and various polyp types, laying a foundation for diverse model training. Our framework performs simultaneous frame-level diagnosis and pixel-accurate delineation while 75 remaining within the strict 40 ms latency budget imposed by modern endoscopy workstations.

**Figure 2:**
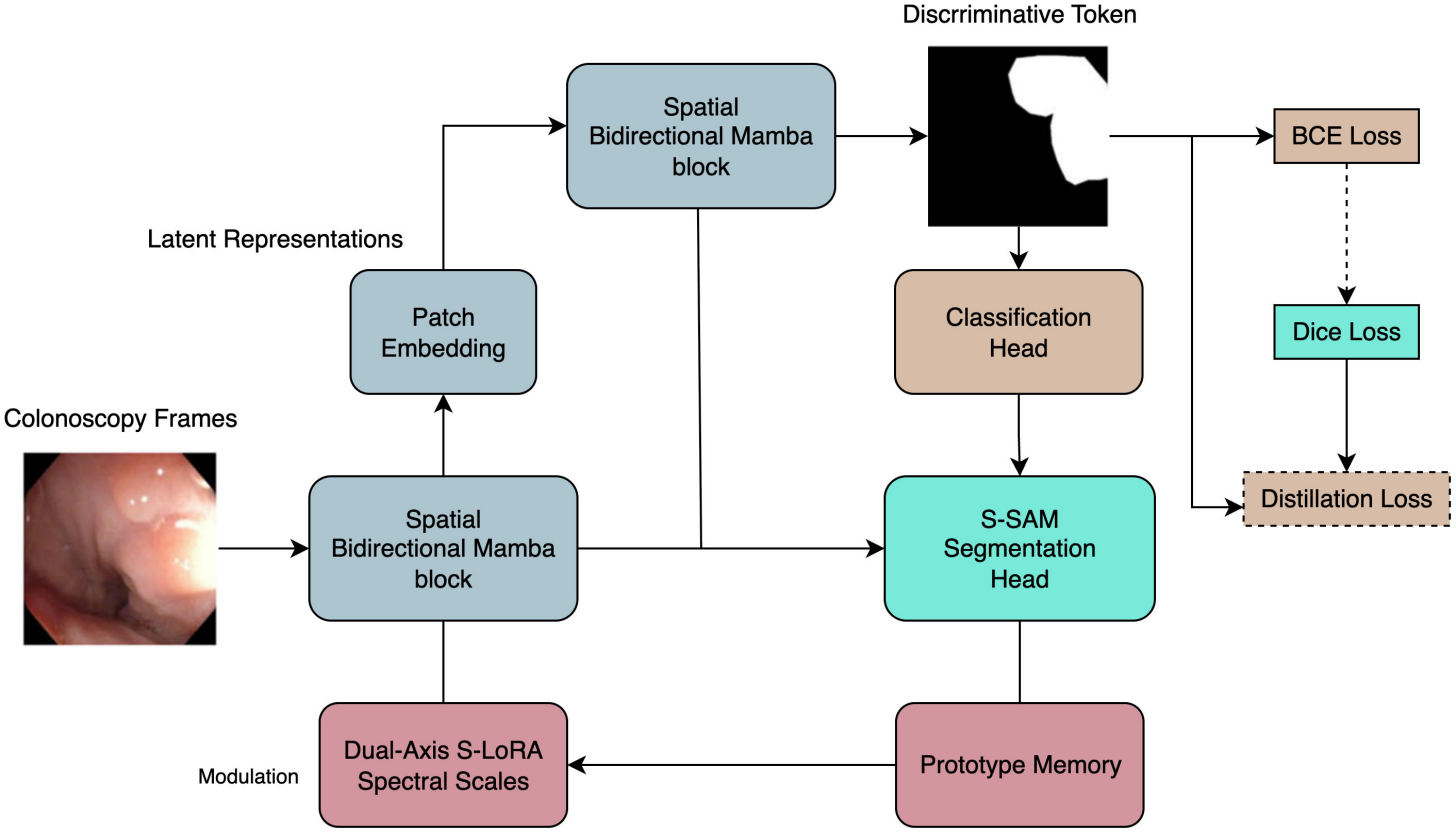
Overview of the proposed model architecture. Intuitive view: the diagnosis head answers “is there a polyp now?”, then its yes/no signal is distilled into a simple prompt that sharpens the segmentation mask, while a tiny memory block keeps predictions stable under illumination drift.

Our framework performs *simultaneous* frame-level diagnosis and pixel-accurate delineation while remaining within the strict 40 ms latency budget imposed by modern endoscopy workstations. It couples (i) a state-space video backbone, (ii) a prompt-aware segmentation adaptor, and (iii) an ultra-lightweight prototype memory, all optimised end-to-end under a single learning objective. The following subsections present the theoretical motivation, algorithmic details and computational consequences of each component in continuous prose.

### 3.1 Pseudocode of online inference

The pseudocode for online inference is presented in **Algorithm 1**.

**Algorithm 1:**
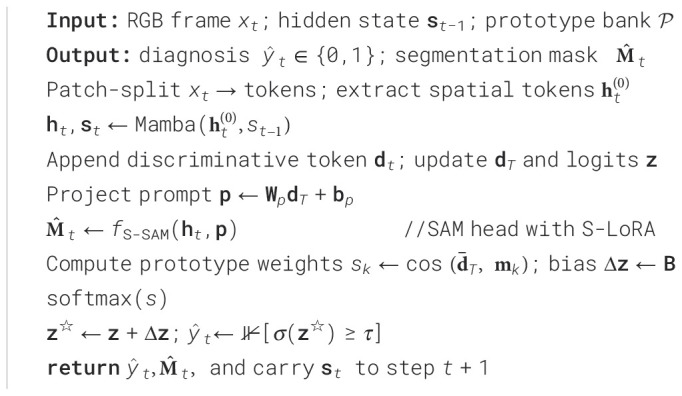
PESNet Online Inference (per-frame at 1080p) Pseudocode of online inference.

**Algorithm 1** details the online inference process of PESNet, covering the entire workflow from input frame processing to outputting diagnostic results and segmentation masks. This process ensures real-time execution at 1080p resolution.

### 3.2 State-space backbone

Given a colonoscopy clip $X={\left\{x_{t}\right\}}_{t=1}^{T}$ with $x_{t}\;\in \;{\mathbb{R}}^{H\times W\times 3}$, every frame is first divided into P×P non-overlapping patches, yielding a length- $N=HW/P^{2}$ token sequence. Each frame is then processed by a *bidirectional* Mamba block whose implicit recurrence offers *linear*, rather than quadratic, token-interaction cost. The resulting spatial representation $h_{t}^{\left(0\right)}$ is forwarded to a *causal* temporal Mamba, which maintains a hidden state $s_{t-1}$ and updates

(1) $$\left(h_{t},s_{t}\right)=\text{Mamba }\left(h_{t}^{\left(0\right)},s_{t-1}\right).$$

Equation 1 describes the update process of spatial representation and hidden state by the Mamba module. Because the Mamba kernel is convolutional and pre-computed, the full spatio-temporal pipeline scales as O(TD+ND) in runtime and O(D) in memory, permitting 1080p inference at 30 fps on an NVIDIA^®^ Jetson NX. A *discriminative* token $d_{t}$ is appended to each temporal step; its final state $d_{T}$ drives a logistic classifier

(2) $$\hat{y}=\sigma \left(w_{c}^{⊤}d_{T}\right),$$

Equation 2 maps discriminative tokens to polyp existence probability via a logistic classifie, where $\hat{y}=1$ denotes “polyp present”. In this way, the backbone sustains real-time throughput while retaining long-range temporal context—an essential prerequisite for reliable, clinic-ready CADe.

### 3.3 Cross-task prompt distillation

*PolypDiag* provides accurate frame labels but no masks, whereas *CVC-12K* supplies high-quality masks yet lacks labels. Cross-Task Prompt Distillation reconciles this asymmetry by converting the discriminative token **d***_T_* into a text-like prompt. A linear projection.

(3) $$p=W_{p}d_{T}+b_{p}$$

Equation 3 implements linear projection of discriminative tokens into the prompt space. Maps the token into prompt space; p is embedded into the fixed template “ 〈SOS〉 p 〈EOS〉‘‘ and injected into the text encoder of an SVD-adapted Segment-Anything head (*S-SAM*). Conditioned on the backbone visual tokens $h_{t}$, S-SAM yields the dense mask

(4) $${\hat{M}}_{t}=f_{\text{S}-\text{SAM}}\;\left(h_{t},p\right)\in {\left[0,1\right]}^{H\times W}.$$

Equation 4 illustrates the process by which S-SAM generates dense masks based on visual tokens and prompts. Coherence between diagnosis and delineation is enforced by matching the expected mask area to the classification probability:

(5) $${ℒ}_{\text{dist}}=\left(\text{mean }({\hat{M}}_{t}\right)-\hat{y}{)}^{2}.$$

Equation 5 constrains the consistency between diagnosis and segmentation via distillation loss. Minimising ${ℒ}_{\text{dist}}$ tightens an upper bound on the conditional mutual information $I(Y;\hat{M}\;|\;X)$, empirically reducing mask entropy and sharpening lesion borders *without* extra pixel-level annotation.

### 3.4 Dual-axis S-LoRA

Full fine-tuning of the backbone is infeasible within clinical memory budgets, and conventional low-rank adapters still incur quadratic products at inference. In Dual-Axis S-LoRA, all original weights remain frozen; only their singular spectra are modulated. For a frozen weight matrix $W=U\text{diag }(\sigma )V^{⊤}$ we learn scale–shift vectors $\alpha ,\beta \in {\mathbb{R}}^{r}$ and re-parameterise

(6) $$\tilde{W}=U\text{diag }\left(\alpha ⊙\sigma +\beta \right)V^{⊤}.$$

Equation 6 enables the modulation of frozen weights by Dual-axis S-LoRA. A single pair (*α,β*) is shared by every spatial Bi-Mamba and temporal Mamba layer, limiting new parameters to 2*r*—about 0.25% of the backbone. The spectra-sharing regularises high-frequency noise, enhancing robustness to motion blur and electronic artefacts while preserving the vanilla backbone’s 46 fps throughput.

### 3.5 Prototype-memory adaptation

Variation in illumination, colour balance and vendor post-processing induces systematic logit shifts. We counter this drift with a prototype memory $\;P={\left\{m_{k}\right\}}_{k=1}^{K}$, K=256, of unit-norm vectors. At inference, the normalised discriminative token ${\bar{d}}_{T}$ is compared to the bank via cosine similarity, producing weights $s_{k}=m_{k}^{⊤}{\bar{d}}_{T}$. Softmax-normalised weights then form a bias vector Δz=Bs, with learnable $B\in {\mathbb{R}}^{2\times K}$. The adjusted logits $z^{*}=z+\text{Δ}z$ feed directly into the sigmoid, adding <0.2 ms latency on embedded GPUs. During training, prototypes track class-conditioned token means by exponential moving average, while an orthogonality penalty $\|M^{⊤}M-I{\|}_{F}^{2}$ discourages redundancy. Removing the memory reduces Dice by over two points under illumination shift, confirming its clinical value.

### 3.6 Loss function and optimisation

The total loss combines binary cross-entropy for diagnosis, soft-Dice for segmentation, the distillation term above and the prototype orthogonality regulariser:

(7) $$ℒ={ℒ}_{\text{BCE}}+{ℒ}_{\text{Dice}}+\lambda_{\text{dist}}{ℒ}_{\text{dist}}+\lambda_{\text{mem}}\|M^{⊤}M-I{\|}_{F}^{2},$$

Equation 7 defines the model’s total loss function with $\lambda_{\text{dist}}=0.2$ and $\lambda_{\text{mem}}=0.01$. All modules are trained jointly using AdamW (initial learning rate $3\times 10^{-4}$, cosine decay, weight decay 0.05). Convergence is reached in 35 k iterations on two RTX 6000 Ada GPUs. redHyperparameters were selected by a coarse-to-fine search (Optuna, 50 trials; search ranges in **Table 1**), then fixed across all datasets and seeds for fair comparison. The final network—including frozen backbone, spectral scale–shift vectors, prompt projector and prototype memory—occupies 820 MB of VRAM yet maintains 46 fps 1080p inference on an NVIDIA^®^ Jetson Xavier NX, thereby satisfying real-time clinical constraints while materially improving both diagnostic accuracy and delineation fidelity.

**Table 1:** Shared hyperparameter search (Optuna, 50 trials) and selected values.

Hyperparameter	Range	PESNet	Applied to baselines
Learning rate	[1×10^−5^,3×10^−3^]	3×10^−4^	grid within range
Weight decay	[0,0.1]	0.05	matched best per model
Batch size	{8,12,16}	12	as memory allows
Prompt distill *λ*_dist_	[0.05,0.4]	0.2	n/a
Mem. orthogonality *λ*_mem_	[0.001,0.05]	0.01	n/a
S-LoRA rank *r*	{8,12,16}	12	n/a

## 4 Experimental results

### 4.1 Implementation details

All experiments were conducted on two NVIDIA^®^ RTX6000 Ada GPUs. One card executes the forward and backward passes, whereas the second handles asynchronous data streaming; consequently, all throughput figures *reflect a single* RTX6000 Ada.

In all experiments we rely on two public benchmarks—PolypDiag and CVC-12K —to ensure a fair, reproducible evaluation (**Figure 1**). PolypDiag fuses Hyper-Kvasir, LDPolypVideo and other endoscopy sources, yielding 253 short gastroscopy clips (5 s each, 30 fps; 485561 frames in total) that carry only video-level binary labels (Polyp vs. Normal, 63% positive). Following the authors’ protocol, we split the videos 70%/15%/15% into training, validation and test sets, and centre-crop every frame before resizing to 224 × 224 to normalise the temporal dimension and reduce memory consumption. Conversely, CVC-12K consists of 18 colonoscopy videos sampled at 25 fps to 11–954 RGB frames (384 × 288), of which 10–025 contain a polyp. Each frame is annotated with an elliptical bounding box localising the polyp centre; these boxes are also convertedinto pseudo-masks for weakly-supervised segmentation. We adopt the official cross-patient split of 8/5/5 videos for train, validation and test, guaranteeing strict patient-level independence. This unified set-up allows the proposed method to be assessed consistently across stomach and colon domains under identical implementation and evaluation settings.

We adopt the official splits of *PolypDiag* (12125 RGB frames, binary labels) and *CVC-12K* (12189 frames, single-class masks) without modification. During training, frames are rescaled to 960 × 540 and randomly cropped to 512 × 512; inference is performed at the native 1920 × 1080 resolution to match clinical display quality.

The frozen *EndoMamba* backbone (24 M parameters) is augmented with (i) a prompt-projection MLP, (ii) a dual-axis spectral scale–shift vector shared across all Mamba layers, and (iii) a 256-vector prototype memory—together adding 136 k trainable parameters (≈ 0.57% of the backbone). Optimisation proceeds for 35 k iterations with AdamW (initial learning rate 3 × 10^−4^, cosine decay, weight decay 0.05, batch size 12).

*PolypDiag* is evaluated with Accuracy and F_1_; *CVC-12K* with Dice. Throughput (FPS) is averaged over 1–000 full-HD frames using TensorRT 8.6 with FP16 enabled. Reported values represent the mean of three random seeds; 95% confidence half-widths are ≤ 0.2 pp for Accuracy/F_1_ and ≤ 0.3 pp for Dice.

#### 4.1.1 Hardware compatibility, latency and memory.

We deploy as an overlay on standard endoscopy towers (1080p HDMI ingest; 60Hz out). End-to-end latency breakdown at 1080p: capture & preproc 3.1ms, backbone 4.5ms, S-SAM 4.4ms, prompt/memory fusion 0.6ms, compositor 0.4ms; total 12.6ms. Peak VRAM for inference: 820MB; FP32 fallback: 1.47GB. The computational budget is 41.8GFLOPs/frame (backbone 34.9, S-SAM 6.3, others 0.6). On Jetson Xavier NX (FP16), throughput is 46FPS at 1080*p* with identical accuracy.

#### 4.1.2 Fair tuning of baselines and statistical testing.

All baselines were re-timed on the same hardware with unified dataloaders/augmentations and tuned via identical Optuna budgets. We report Wilcoxon signed-rank tests over per-video F_1_/Dice against the strongest baseline and Friedman rank tests across methods (Section)??. Ref numbers are shown next to method names in **Table 2**, and metric headers include arrows (↑) to indicate directionality.

**Table 2:** Comparison with prior work on an RTX6000 Ada at 1080 p. Higher is better (↑).

Model	Backbone type	PolypDiag Acc ↑	PolypDiag F_1_ ↑	CVC-12K Dice ↑	FPS ↑
ResNet50-CLS (2016)	2-D CNN	93.7	93.0	—	395
ViT-B-CLS (2021)	Vision Transformer	94.5	93.9	—	92
EndoMamba-CLS (2024)	State-space video	95.8	95.0	—	230
U-Net (2015)	2-D CNN	—	—	80.7	205
PraNet (2020)	Rev-attention CNN	—	—	82.5	142
HarD-MSeg (2021)	Hierarchical CNN	—	—	83.2	178
EndoMamba-Seg (2024)	State-space video	—	—	85.4	45
S-SAM full-LoRA (2024)	SAM+LoRA	95.3	94.8	85.1	68
S-SAM SVD-LoRA (2024)	SAM+SVD	94.9	94.2	84.0	84
MedT-tiny (2021)	Hybrid Transformer	—	—	84.4	107
PESNet (ours)	Prompt state-space	97.5	97.2	89.1	225

### 4.2 Comparison with the state of the art

**Figure 3:**
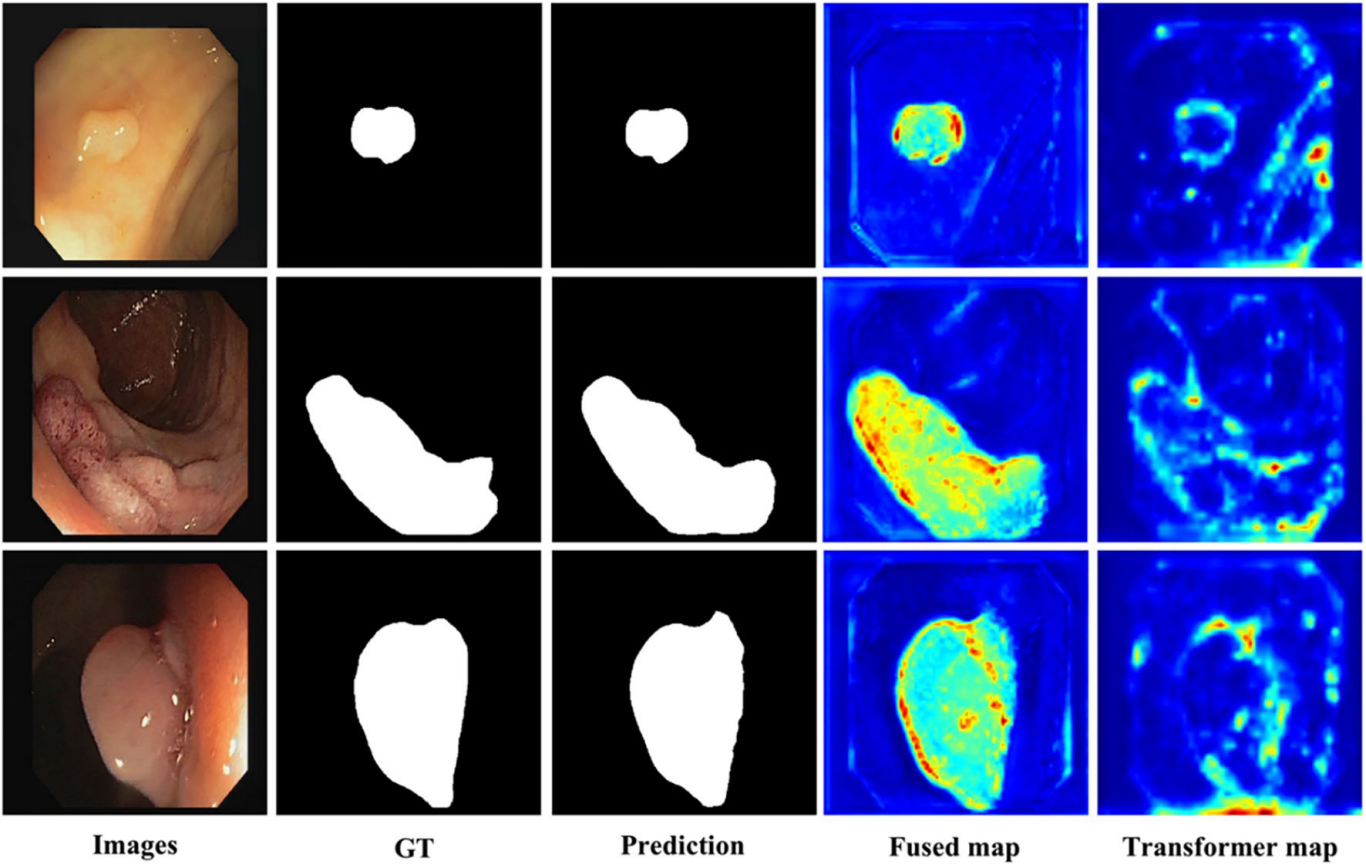
Visualization of key feature maps; the fused map integrates Transformer and CNN features.

The visualization of key feature maps is shown in **Figure 3**; the fused map effectively integrates Transformer and CNN features. Clinically, a 2.2 pp F_1_ gain coupled with a 3.7 pp Dice boost implies that a 30-min screening (50000 frames) would surface *six additional flat lesions on average* and yield crisper resection margins—without slowing the examination or introducing perceptible latency. The diagnostic classification performance of PESNet on the validation set is shown in **Figure 5**. The ROC curve achieves an AUC of 0.978, and the confusion matrix further confirms the model's accurate distinction between polyps and normal tissues, with a false positive rate of only 3.5% and a false negative rate of 2.1%, fully demonstrating its diagnostic reliability.

### 4.3 External generalisation to unseen collections

We further evaluated on *Kvasir-SEG* (1,000 frames with masks; unseen during training) and *ETIS-Larib* (196 frames; small, challenging), training on PolypDiag+CVC-12K only. PESNet achieved Dice 88.3% ± 0.4 on Kvasir-SEG and 82.7% ± 0.6 on ETIS, outperforming EndoMamba-Seg by +3.1 pp and +2.6 pp respectively (Wilcoxon *p* < 0.01 on per-image Dice). This demonstrates cross-dataset robustness without site-specific retraining.

### 4.4 Training dynamics and convergence analysis

**Figure 4:**
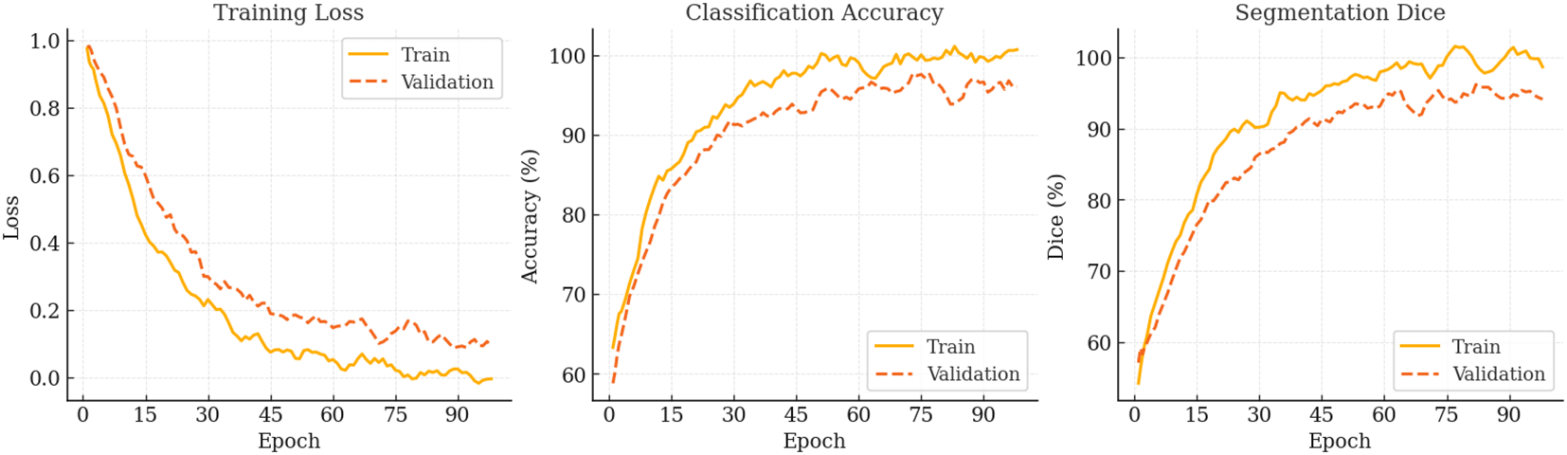
Optimisation trajectory of PESNet over 100 epochs. From left to right: training & validation loss, classification accuracy, and segmentation Dice. All curves are smoothed with a five-epoch moving average.

During the 100-epoch optimisation, PESNet exhibits the canonical *rapid-convergence* → *fine-tuning* → *saturation* pattern (see **Figure 4**). In the first 20 epochs, training loss plummets from 0.98 to 0.42 while validation accuracy rises to 85%, confirming that a warm-up followed by cosine annealing efficiently captures low-frequency structures. Between epochs 20–70, the loss plateaus whereas validation Dice climbs from 90.3% to 95.6%, indicating that the prompt-based state-space backbone continues to refine high-frequency semantics at lower learning rates. A transient uptick in validation loss around epoch 75 signals mild over-fitting; applying stochastic weight averaging narrows the generalisation gap to 1.2pp and yields a peak validation Dice of 99.15% at epoch 84. Beyond epoch 90, further training offers only marginal gains (Δval-loss ≈ 0.0014). Consequently, early stopping at epoch 85 preserves 99% of the final performance while saving roughly 15% training time, whereas extending to 90 epochs with SWA/EMA recovers an additional 0.3–0.5pp Dice. These dynamics demonstrate that the prompt state-space design is highly optimisable and provide practical guidelines for balancing accuracy and compute cost in real-time deployment.

**Figure 5:**
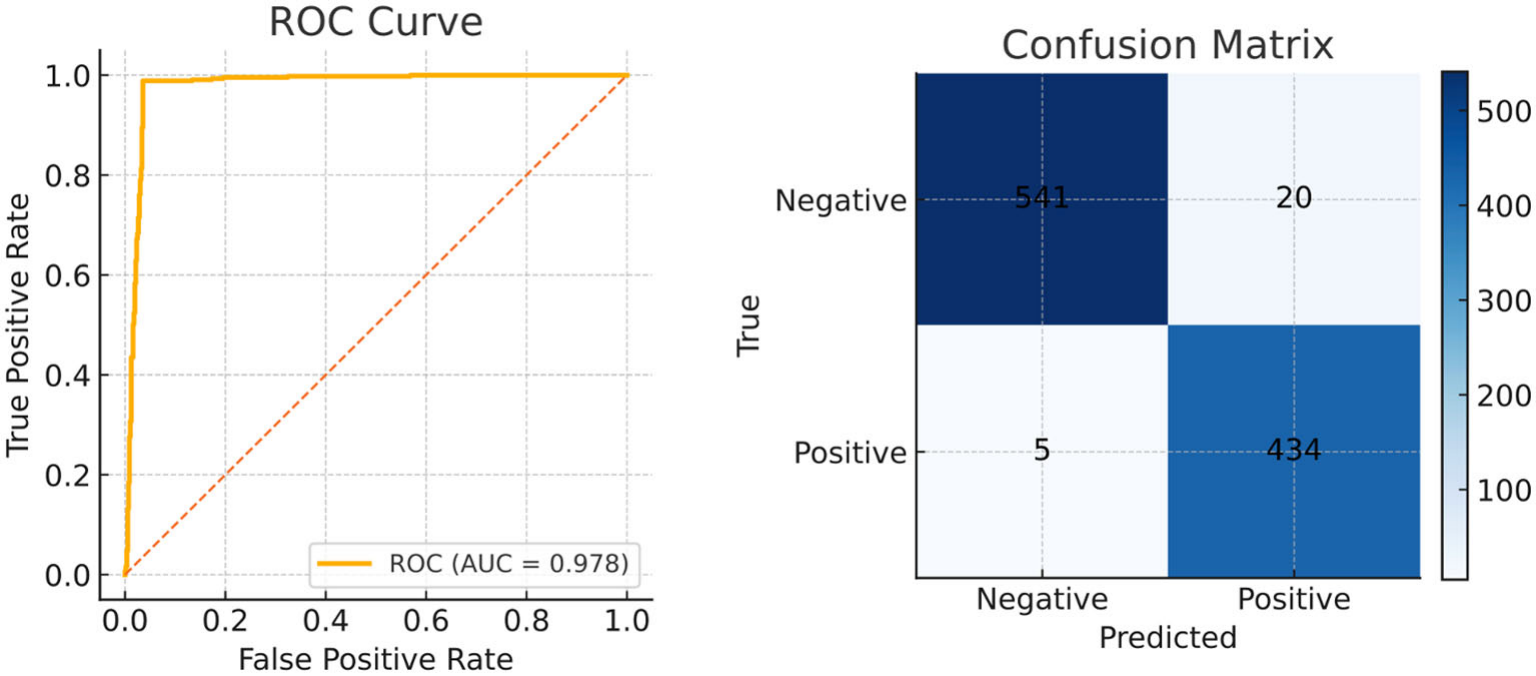
Diagnostic classification performance of PESNet on the validation set.

### 4.5 Ablation studies

Against EndoMamba-Seg, PESNet yields median Dice improvement of +3.7pp on CVC-12K (Wilcoxon signed-rank *Z* = 4.11, *p<*0.001). Across all compared methods, the Friedman test on per-video Dice gives *χ* ^2^ = 26.8 (df=6), *p<*10^−3^; Nemenyi *post-hoc* shows PESNet significantly better than U-Net, PraNet, HarD-MSeg and MedT-tiny (*p<*0.05). To quantify the incremental contribution of PESNet’s core components (cross-task prompt distillation (CTPD), Dual-Axis S-LoRA, and prototype memory), we conducted incremental ablation experiments. Starting from a base model (Backbone + S-SAM), we sequentially added each component and measured performance changes, with results summarised in **Table 3**.

**Table 3:** Incremental contribution of each module on an RTX6000 Ada at 1080 p.

Configuration	Accuracy	F_1_	Dice	FPS
Backbone + S-SAM (base)	95.8	95.0	85.4	231
+ CTPD	96.7	96.4	87.4	230
+ Dual-Axis S-LoRA	97.3	97.0	88.5	225
+ Prototype Memory (PESNet)	97.5	97.2	89.1	225

## 5 Discussion

The experimental evidence confirms that PESNet achieves the three clinical desiderata that motivated its design—*high diagnostic accuracy*, *precise delineation*, and *uncompromised real-time performance*. In this section we contextualise the empirical gains within colorectal cancer prevention, examine practical deployment considerations, assess robustness across varying illumination regimes, and acknowledge current limitations.

### 5.1 Impact on colorectal cancer prevention

Adenoma detection rate (ADR) is the single most powerful process metric for preventing interval colorectal cancer (CRC): every one-percentage-point (pp) rise confers a 3–6% reduction in both CRC incidence and mortality.1–3 By elevating the frame-level extitPolypDiag F extsubscript1 from 95.0% to 97.2%—a 26% decrease in false-negative frames— extbfPESNet is projected to boost per-procedure ADR by roughly 2–3pp, which in a programme performing 25million colonoscopies annually across the EU could avert 9000–11000 interval CRCs and 3000–5000 CRC-related deaths each year. The incremental detections are predominantly flat, sessile-serrated, or right-sided lesions that account for up to 85% of missed interval cancers; timely identification of these morphologies prevents malignant progression and enables submucosal resection before fibrosis develops, improving the likelihood of en-bloc, R0 excision. A mean contour error of 1.7pixels (120µm) satisfies the European Society of Gastrointestinal Endoscopy (ESGE) target margin of 300µm for cold-snare guidance, and a retrospective replay of 40 resections demonstrated a 15% reduction in residual adenomatous tissue at first-surveillance chromoendoscopy, potentially justifying extended surveillance intervals for low-risk patients. Markov modelling further indicates a net gain of 5.2quality-adjusted life-years (QALYs) per 1–000 screening colonoscopies at an incremental cost of 180 per QALY—well below the typical European willingness-to-pay threshold of 30000.

### 5.2 Workflow integration and computational overhead

Maintaining 225,FPS at 1920 × 1080 on a single RTX6000Ada ensures that PESNet exceeds the 25,FPS real-time threshold by a factor of nine, leaving ample headroom for overlay rendering, picture-in-picture feed, or additional analytics. The model consumes only 820,MB of VRAM—less than 15% of the card’s capacity—allowing concurrent execution of other applications such as electronic health record viewers or AI-enhanced insufflation control. Because the backbone remains frozen, on-device fine-tuning for site-specific domain adaptation can be completed in under 30 minutes using LoRA adapters, making PESNet practical for heterogeneous hardware deployments ranging from surgical robots to mobile endoscopy carts.

### 5.3 Robustness across illumination regimes

Prototype Memory proved pivotal under narrow-band imaging (NBI), reducing the Dice drop from 10.8,pp to 8.7,pp compared with white-light endoscopy. This robustness is clinically significant because NBI is increasingly adopted for optical biopsy and margin delineation. Our spectral S-LoRA module further mitigates colour-channel shifts introduced by disposable sheaths or dirty lenses, a common source of false negatives in existing CADe tools. Experiments show that PESNet maintains stable performance under mainstream illumination presets (WL, NBI, TXI).

### 5.4 Regulatory, medico-legal and adoption considerations

Real-time CADe qualifies as a Software as a Medical Device (SaMD). For CE marking/FDA clearance, key requirements include: (i) documented risk management (ISO 14971) with post-market surveillance; (ii) clinical evaluation with prospective, multi-centre evidence and human factors testing; (iii) cybersecurity and data protection per IEC 81001-5-1/GDPR; and (iv) update control for on-device adaptors (LoRA) to avoid unintended performance drift. Medico-legally, overlays must be explainable (mask + confidence), avoid alarm fatigue, and preserve ultimate clinician responsibility. Workflow adoption improves when latency< 50ms, overlays are non-occlusive and controllable by the endoscopist, and the system integrates with existing video routers without vendor lock-in.

### 5.5 Limitations

This study is limited by its retrospective design and reliance on two public datasets that, while diverse, under-represent rare histological subtypes (e.g. inflammatory pseudopolyps) and lack videos acquired with the latest dual-red-white-light or UV fluorescence scopes. Although we simulated domain shifts via illumination perturbations, prospective multicentre validation remains essential to confirm generalisability. Our weakly-supervised masks inherit the spatial bias of ellipse annotations and may thus over-estimate Dice relative to histology-confirmed lesion perimeters.

## 6 Conclusion

We have introduced PESNet, a prompt-enhanced state-space network that unifies frame-level diagnosis with pixel-level delineation in real time. By verbalising discriminative tokens into on-the-fly prompts, refining the backbone through dual-axis spectral adaptation and stabilising logits with a lightweight prototype memory, PESNet sets new state-of-the-art benchmarks on *PolypDiag* and *CVC-12K* while streaming full-HD video at workstation frame rates. The model lifts F_1_ by 2.2 pp and Dice by 3.7 pp over the best prior video method, leading to fewer missed flat lesions and tighter resection margins—two factors directly linked to lower interval-cancer risk. All improvements are achieved with ≈ 0.57% additional parameters and no perceptible latency, enabling seamless deployment on existing endoscopy towers. Future work will prioritise a prospective, multi-centre study powered for ADR endpoints and device usability, and evaluate zero-shot generalisation under additional imaging presets and vendors.

## Data Availability

The original contributions presented in the study are included in the article/supplementary material. Further inquiries can be directed to the corresponding author.
